# Management of esophageal stricture after complete circular endoscopic submucosal dissection for superficial esophageal squamous cell carcinoma

**DOI:** 10.1186/1471-230X-11-46

**Published:** 2011-05-04

**Authors:** Hajime Isomoto, Naoyuki Yamaguchi, Toshiyuki Nakayama, Tomayoshi Hayashi, Hitoshi Nishiyama, Ken Ohnita, Fuminao Takeshima, Saburo Shikuwa, Shigeru Kohno, Kazuhiko Nakao

**Affiliations:** 1 Department of Endoscopy, Nagasaki University Hospital, 1-7-1 Sakamoto, Nagasaki 852-8102, Japan; 2 Department of Pathology, Nagasaki University Hospital, 1-7-1 Sakamoto, Nagasaki 852-8102, Japan; 3 Department of Gastroenterology and Hepatology, Nagasaki University Hospital, 1-7-1 Sakamoto, Nagasaki 852-8102, Japan

## Abstract

**Background:**

Endoscopic submucosal dissection (ESD) permits removal of esophageal epithelial neoplasms *en bloc*, but is associated with esophageal stenosis, particularly when ESD involves the entire circumference of the esophageal lumen. We examined the effectiveness of systemic steroid administration for control of postprocedural esophageal stricture after complete circular ESD.

**Methods:**

Seven patients who underwent wholly circumferential ESD for superficially extended esophageal squamous cell carcinoma were enrolled in this study. In 3 patients, prophylactic endoscopic balloon dilatation (EBD) was started on the third post-ESD day and was performed twice a week for 8 weeks. In 4 patients, oral prednisolone was started with 30 mg daily on the third post-ESD day, tapered gradually (daily 30, 30, 25, 25, 20, 15, 10, 5 mg for 7 days each), and then discontinued at 8 weeks. EBD was used as needed whenever patients complained of dysphagia.

**Results:**

*En bloc *ESD with tumor-free margins was safely achieved in all cases. Patients in the prophylactic EBD group required a mean of 32.7 EBD sessions; the postprocedural stricture was dilated up to 18 mm in diameter in these patients. On the other hand, systemic steroid administration substantially reduced or eliminated the need for EBD. Corticosteroid therapy was not associated with any adverse events. Post-ESD esophageal stricture after complete circular ESD was persistent, requiring multiple EBD sessions.

**Conclusions:**

Use of oral prednisolone administration may be an effective treatment strategy for reducing post-ESD esophageal stricture after complete circular ESD.

## Background

Endoscopic submucosal dissection (ESD) was developed to dissect directly along the submucosal layer for gastric epithelial neoplasia [1,2]. ESD has an advantage over endoscopic mucosal resection (EMR) for removing gastrointestinal tumors *en bloc*, regardless of their size [1,2]. ESD allows precise histological assessment of the specimens excised in one piece with tumor-free lateral/basal margins, preventing residual disease and local recurrence [2,3].

Several studies in advanced institutes showed that ESD was promising for superficial esophageal epithelial neoplasms with nominal risks for nodal or distant metastasis [4,5]. Nevertheless, a substantial risk of procedure-related complications, including potentially life-threatening perforation and postprocedural stenosis, have been reported [4]. Although the exact incidence of luminal stricture after esophageal ESD is unknown, it is related to the extent of the circumference being resected [6]. In particular, esophageal stricture occurs commonly following ESD involving the entire circumference of the esophageal lumen [6]. The resultant dysphagia substantially decreases patients' quality of life (QOL), requiring multiple sessions of endoscopic balloon dilatation (EBD) [7].

Although EBD has been a treatment of choice in the setting of benign esophageal strictures [8], intralesional steroid injection into corrosive or anastomotic strictures can achieve remission of dysphagia or reduce the need for repeated EBD [9,10]. However, certain patients with recalcitrant esophageal stricture do not respond to local steroid therapy, although systemic steroid administration often resolves the problem in such cases [11,12]. We report on 7 consecutive patients with superficial esophageal squamous cell carcinoma (SCC) who underwent wholly circumferential ESD. Among these patients, 4 were treated with an 8-week course of oral prednisolone, which effectively either prevented esophageal stricture or reduced the number of EBD sessions.

## Methods

### Patients

A total of 7 patients (6 men, 1 woman; mean age, 68 years; range, 60-74 years) underwent complete circular ESD for superficially extended SCC of the esophagus at Nagasaki University Hospital. The clinicopathological features of each patient are summarized in Table 1. The Ethics Committee of Nagasaki University Hospital approved this study, and written informed consent was obtained from each patient. The tumor was demarked using chromoendoscopy with iodine staining. Because all superficial SCCs involved nearly the entire circumference of the esophageal lumen, complete circular resection was considered necessary in each patient.

**Table 1:** Clinicopathological features and treatment measures for esophageal stricture for 7 patients with esophageal squamous cell carcinoma treated by complete circular endoscopic submucosal dissection

**Case No**.	Age	Endoscopic morphology	Resection size (mm)	Tumor size (mm)	Thoracic location	Depth of tumor invasion	Treatment for stricture	EBD times
1	73	IIc	43	40	Lower	m2	Preemptive EBD	30
2	60	IIb	70	56	Middle	sm1	Preemptive EBD	20
3	65	IIb	94	67	Middle	m2	Preemptive EBD	48
4	72	IIb	70	70	Middle	m2	Prednisolone	2
5	74	IIc	75	65	Middle	m2	Prednisolone	0
6	66	IIb	81	58	Middle	sm1	Prednisolone	11
7	65	IIc	80	65	Midddle	m2	Prednisolone	0

m2, intramucosal invasive carcinoma limited to the lamina propria mucosae; sm1, slightly invasive carcinoma into the submucosa (<200 μm); EBD, endoscopic balloon dilatation.

### ESD

For the ESD procedure, a single-channel upper gastrointestinal endoscope with a water-jet system (GIF-Q260J, Olympus Medical Systems, Tokyo, Japan) was used with a transparent cap attached to the endoscope tip. Circumferential markings were made on the proximal and distal sides of the normal-appearing mucosa at least 5 mm from the lesions. A high-frequency generator (VIO300D; ERBE Elektromedizin GmbH, Tübingen, Germany) was set at the forced coagulation mode (Effect 2, output 40 W) to incise the mucosa. A ready-to-use hyaluronic acid solution (MucoUp, Seikagaku Co., Tokyo, Japan) was injected into the submucosal layer to lift the surrounding mucosa. The distal mucosal incision was completed first all the way around the markings with the FlushKnife (Fujinon-Toshiba ES System Co, Omiya, Japan) under the Endo cut I mode (Effect 4, Duration 2, Interval 3). The proximal mucosa was then incised in the same circular fashion. The submucosa was incised using the FlushKnife under the forced coagulation mode (Effect 2, output 40 W), starting beneath the proximal mucosal incision in each quadrant. The submucosal dissection was advanced to reach the distal side of the mucosal incision, thus creating a submucosal tunnel. Then, the submucosa between the 4 longitudinal tunnels was dissected to connect together until the lesion was detached. To control bleeding during ESD or to prevent possible bleeding from visible vessels in the artificial ulcer immediately after resection, a hemostatic forceps (Coagrasper, Olympus) was used in the soft coagulation mode (Effect 5, 60 W). Each patient was sedated by intravenous injection of 7.5 mg diazepam and 50 mg pethidine, and additional diazepam (2.5 mg) or pethidine (25 mg) was given alternately as needed for conscious sedation throughout ESD. CO_2 _insufflation was used during the procedure. Procedure-related bleeding after ESD was defined as bleeding that required transfusion or surgical intervention or bleeding that caused the hemoglobin level to fall by 2 g/dL [13]. Procedure-related perforation was diagnosed endoscopically or by the presence of free air on a plain chest X-ray and chest computed tomogram (CT) [5]. Longitudinal diameter was measured from the oral edge to the anal edge of resected specimens that were stretched appropriately. The tumor longitudinal diameter was also documented. Excised specimens were fixed in 10% buffered formalin, paraffin-embedded, sectioned perpendicularly at 2-mm intervals, and stained with hematoxylin and eosin. *En bloc *resection refers to resection in one-piece [14]. Follow-up endoscopy with iodine staining and contrast-enhanced CT of the cervix, thorax, and abdomen were scheduled at 3, 6, and 12 months after ESD and then annually thereafter. Biopsy specimens during each follow-up endoscopy were taken from any mucosal abnormalities to assess the presence of local recurrent tumor.

### Postprocedural esophageal strictures and management

Between June 2008 and September 2009, pre-emptive EBD using a CRE balloon (Boston Scientific Co., Boston, MA, USA) was started in 3 patients (Case No. 1-3, Table 1) on the third post-ESD day and performed twice a week for 8 weeks. The postprocedural stricture was dilated up to 18 mm in diameter. In cases of persistent dysphagia, EBD was performed until dysphagia resolved. Since October 2009, oral prednisolone was administered to 4 patients (Case No. 4-7, Table 1), starting at a dose of 30 mg daily on the third post-ESD day, tapered gradually (daily 30, 30, 25, 25, 20, 15, 10, 5 mg for 7 days each), and then discontinued 8 weeks later. EBD was applied as needed whenever the patients complained of dysphagia.

### Statistical analysis

The statistical significance of data was determined using the Student's t test. A P value less than 0.05 was considered statistically significant.

## Results

As shown in Table 1, there were 4 IIb and 3 IIc macroscopic types in accordance with the Paris endoscopic classification [15]. They were located in the middle (6) and lower (1) thoracic esophagus. All lesions were resected in an *en bloc *fashion and removed with tumor-free lateral and basal margins. There was no procedure-related bleeding or perforation. The mean operation time was 163 minutes (range, 100 to 260 minutes). The overall longitudinal diameter of resected specimens was 73.3 mm (range, 43 to 94 mm). The overall mean longitudinal diameter of the excised tumors was 60.1 mm (range, 40 to 70 mm). As for tumor invasion depth, there were 5 m2 lesions (intramucosal invasive carcinoma limited to the lamina propria mucosae) and 2 sm1 lesions (slightly invasive carcinoma into the submucosa (<200 μm) that were well-differentiated SCC with expanding tumor growth lacking angiolymphatic invasion. The 2 patients with submucosally invasive SCC underwent chemoradiation, and local recurrence and distant metastasis were not observed on a systemic CT scan 7 and 20 months later. All patients were still alive during the mean follow-up period of 11 months (range, 5 to 22 months).

**Figure 1:**
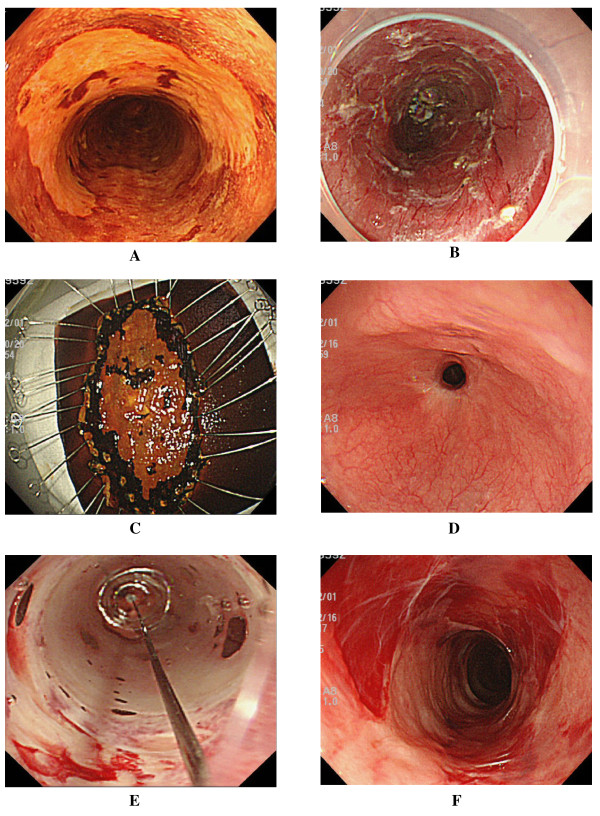
**In Case 3, complete circular endoscopic submucosal dissection (ESD) was achieved, and endoscopic balloon dilatation (EBD) was performed preemptively**. Nevertheless, he required total 48 sessions to relieve his dysphagia. **A**. Chromoendoscopy with an iodine solution reveals the iodine-unstained area spreading to involve nearly the entire circumference of the esophagus (Case 3, Table 1). Wholly circumferential ESD was performed. **B**. Artificial ulcer immediately after complete circular resection. **C**. The tumor was removed *en bloc *with tumor-free lateral and basal margins, and histopathological assessment revealed intramucosal invasive squamous cell carcinoma (m2). Repeat esophagoscopy revealed persistent esophageal stricture (**D**) despite 16 sessions (twice a week, for 8 weeks) of EBD (**E**), which was started on the third postoperative day. Temporary improvement of the stricture was achieved with EBD (**F**), but this patient required 48 EBD sessions.

**Figure 2:**
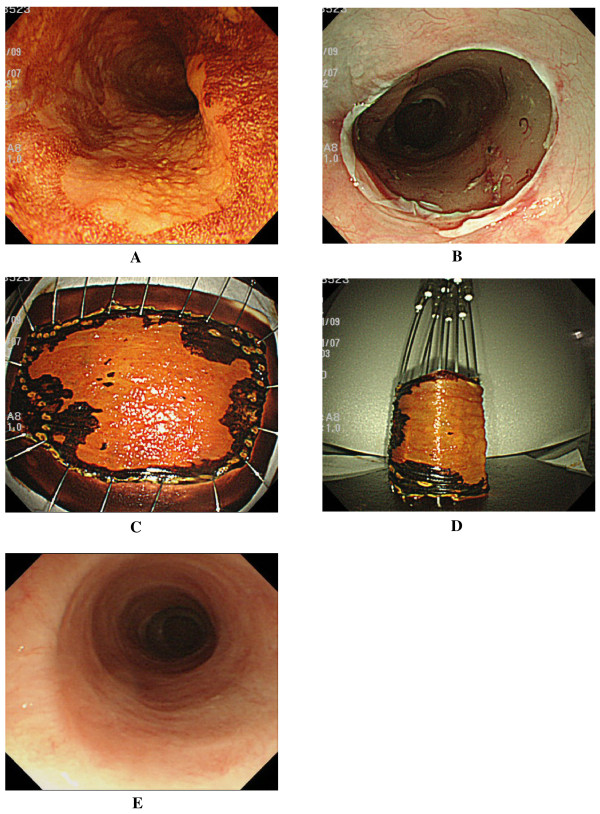
**In Case 5, complete circular ESD was achieved, and oral prednisolone was given**. He has not required any EBD sessions without no postprocedural stricture and the related dysphagia. **A**. Chromoendoscopy with iodine staining revealed a discolored area spreading to involve nearly the entire circumference of the esophagus in the middle thoracic esophagus (Case 5, Table 1), and wholly circumferential, endoscopic submucosal dissection was performed. **B**. Artificial ulcer immediately after complete circular resection. Complete circular resection was achieved (**C**), and the tumor was removed *en bloc *with tumor-free lateral and basal margins (**D**). Histopathological assessment revealed intramucosal invasive squamous cell carcinoma (m2). Oral prednisolone (30 mg) was initiated on the third postoperative day, tapered, and then discontinued 8 weeks later. **E**. Follow-up endoscopy 6 months later revealed no postprocedural stricture without EBD.

In the prophylactic EBD group, postprocedural stricture accompanied by dysphagia occurred in all 3 patients. These patients required multiple EBD sessions (mean 32.7 sessions, Table 1). In particular, Case 3 required 48 sessions to relieve his dysphagia (Figure 1). On the other hand, the 4 patients who received 8-week systemic steroid treatment required fewer EBD sessions (Table 1). There was a significant difference in the number of required EBD sessions between the two treatment groups (p < 0.05, Student's t test). There were 2 patients who did not experience luminal stricture and dysphagia; thus, EBD was not done in all patients who received steroid therapy (Figure 2). No adverse events related to oral prednisolone administration were seen in any patient.

## Discussion

Esophageal ESD is associated with a substantial risk of complications [4,5]. Because relatively large superficial esophageal epithelial neoplasms are candidates for this procedure, postoperative stricture has become a major concern with regard to long-term clinical outcome [6]. Esophageal strictures can cause dysphagia and result in decreased QOL or aspiration pneumonia [3]. Once the patient has started to experience dysphagia due to esophageal stricture, endoscopic dilatation with either a bougie or a balloon (EBD) is the preferred mode of treatment irrespective of the etiology [8]. Ono *et al*. reported that post-ESD stricture with dysphagia was successfully managed with EBD in a median of 2 sessions [6]. Again, repetitive, periodic EBD was effective in controlling and preventing postprocedural stricture even after semicircular esophageal ESD [7]. However, a subset of patients with esophageal stricture do not achieve symptomatic relief despite repeated EBD. Compared with peptic stricture, corrosive stricture may be long, tortuous, and even multiple. Furthermore, corrosive strictures may require more esophageal dilatation sessions, which are often prolonged; these type of strictures are also more likely to recur [16]. Radiation-induced stricture also falls in the treatment-resistant category [17]. Our experience with the current series indicates that complete circular ESD for extended esophageal SCC can cause persistent esophageal stricture. Indeed, pre-emptive EBD was started on the third post-ESD day and repeated twice a week for no less than 2 months, but each patient required multiple EBD sessions due to persistent dysphagia.

There are other ways to treat esophageal stenosis, such as temporary stent insertion and local corticosteroid injection [9,18]. Prior studies suggest the usefulness of intralesional steroid injection into benign esophageal strictures for augmentation of the effects of endoscopic dilatation [9,10]. However, there are some cases with recalcitrant esophageal stricture despite intralesional steroid injection followed by endoscopic dilatation [11,12]. In addition, potential risks for esophageal perforation and mediastinitis or pleural effusion are associated with local therapy [10]. It would be riskier when injecting directly onto the ulcer bed immediately after esophageal ESD. Recently, Morikawa *et al*. described several cases with persistent esophageal stricture that were resistant to intralesional steroid injection but were successfully managed by systemic steroid administration [11]. Similarly, there was a case report on successful treatment of severe refractory anastomotic stricture after esophageal atresia repair by EBD combined with systemic steroid administration [12]. There could also be a risk of esophageal candidiasis, inasmuch as there is a propensity for this in stenotic esophageal lesions; corticosteroid administration could further increase the risk [10].

In the present study, 4 patients were given oral prednisolone, which was effective in controlling postprocedural stricture and preventing repeat EBD. It was started at a relatively moderate dosage of 30 mg and continued for only 8 weeks. Accordingly, the total corticosteroid dose was nealy 1000 mg for the treatment course, and there were no adverse events. The underlying mechanisms whereby systemic prednisolone may control post-ESD stricture and reduce EBD sessions remain unclear. Corticosteroids can inhibit not only collagen synthesis but also enhance collagen breakdown, thereby inhibiting stricture formation [10]. It is postulated that triamcinolone can prevent the cross-linking of collagen that results in scar stricture [19]. Recently, esophageal strictures have been managed using new technologies, such as biodegradable stents [18] or autologous oral mucosal sheets [20]. However, until these methods are available in the clinical setting, our experience with systemic prednisolone administration for complete circular ESD may thus offer an alternative option to decrease the risk of benign esophageal strictures and reduce the number of EBD sessions.

## Conclusions

Complete circular esophageal ESD was safely and successfully achieved in our small series with superficially extended SCC. Oral prednisolone administration, but not prophylactic EBD alone, was effective in controlling the post-ESD stricture and in preventing repeated EBD even after complete circular ESD. Further evaluation in a larger series is needed before this therapeutic option can be widely accepted.

## Competing interests

The authors declare that they have no competing interests.

## Authors' contributions

HI, acquisition of data, analysis and interpretation of data, conception and design, drafting the manuscript; NY, acquisition of data, analysis and interpretation of data; TN, acquisition of data and analysis and interpretation of data; TH; acquisition of data and analysis and interpretation of data; HN, acquisition of data and analysis and interpretation of data; KO, analysis and interpretation of data; FT, drafting and revising the manuscript; SS, analysis and interpretation of data; SK, drafting and revising the manuscript; KN, drafting and revising the manuscript, and general supervision of the research group. All authors read and approved the final manuscript.

## Pre-publication history

The pre-publication history for this paper can be accessed here:

http://www.biomedcentral.com/1471-230X/11/46/prepub
